# Multi-gene Co-expression systems in *E. coli*: From single-vector designs to programmable expression platforms

**DOI:** 10.1016/j.synbio.2025.12.008

**Published:** 2026-01-10

**Authors:** Rui Liu, Lu-Wei Wang, Zi-Han Gao, Xiao-Tong Sun, Shu-Ran Lv, Huan Liu, Sa-ouk Kang, Bo Sun

**Affiliations:** a School of Food Engineering, Yantai Engineering Research Center of Food Green Processing and Quality Control, Ludong University, Yantai, Shandong, 264025, PR China; b Laboratory of Biophysics, School of Biological Sciences, and Institute of Microbiology, Seoul National University, Seoul, 151-742, Republic of Korea; c State Key Laboratory of Digital Medical Engineering, Southeast University, Nanjing, 210096, PR China

**Keywords:** *E. coli*, Co-expression systems, IRES, 2A peptides, Synthetic biology, Programmable expression

## Abstract

*Escherichia coli* (*E. coli*) has long served as a versatile workhorse for recombinant protein production. As synthetic biology expands the demand for coordinated expression of multiple genes, co-expression systems in *E. coli* have evolved from basic dual-gene constructs to programmable, polygenic expression platforms. This review critically examines the major strategies enabling multigene co-expression in *E. coli,* including internal ribosome entry sites (IRES), 2A self-cleaving peptides, dual-promoter cassettes, multicistronic operons, and multi-plasmid configurations. We highlight the mechanistic principles, design trade-offs, and regulatory bottlenecks associated with each approach, such as translational imbalance, inclusion body formation, and plasmid compatibility. Real-world applications in metabolic engineering, complex protein assembly, and biomanufacturing are analyzed to demonstrate the functional advantages of these systems. Finally, we explore emerging programmable toolkits that integrate modular architecture, expression modeling, and AI-assisted design, paving the way for next-generation synthetic expression control in microbial chassis. This review offers a comprehensive and strategic roadmap for researchers engineering multi-gene systems in *E. coli* and beyond.

## 1 Introduction

*Escherichia coli* (*E. coli*) has long been a cornerstone of biotechnology and synthetic biology, valued for its well-characterized genetics, rapid growth, cost-effective cultivation, and high productivity in recombinant protein expression [1]. Decades of development have established robust methods for expressing single genes in *E. coli*, underpinning advances in areas from industrial enzyme production to biomedical research. However, the growing ambitions of metabolic engineering and synthetic biology increasingly demand the coordinated expression of multiple genes in a single host cell [2]. Multi-gene expression (co-expression) enables the assembly of entire biosynthetic pathways, the production of multi-subunit protein complexes, and the co-production of helper factors (e.g., chaperones or foldases) to enhance folding and solubility of target proteins [3]. This represents a paradigm shift from traditional monogenic (single-gene) systems to polygenic expression platforms capable of orchestrating complex genetic programs within *E. coli*.

Co-expression in *E. coli* can be defined as the simultaneous expression of multiple heterologous genes in the same host organism [4]. Achieving reliable co-expression is non-trivial, as it requires carefully engineered genetic architectures and balanced expression to ensure that all target proteins are produced at appropriate levels. Early approaches often relied on monogenic plasmids—multiple plasmids each carrying a single gene—introduced sequentially into one strain [5]. Co-maintenance of multiple plasmids generally requires mutually compatible, distinct origins of replication (replicons) together with different antibiotic selection markers to prevent incompatibility and plasmid loss. As the number of plasmids increases, transformation efficiency and growth rate typically decline, whereas metabolic burden and antibiotic selection pressure rise, for example, the ColE1/p15A/pSC101 combination is commonly employed. Moreover, co-residence of plasmids sharing the same replicon can be transiently stabilized under strong selection but is prone to loss during extended cultivation. Multi-plasmid strategies can be labor-intensive and impose a heavy burden on the cell. New polygenic systems have since been developed that enable multiple genes to be expressed from a single genetic construct or a coordinated set of vectors. These innovations are accelerating progress in metabolic pathway engineering, protein complex reconstitution, and other advanced applications of *E. coli* in biotechnology.

**Fig. 1:**
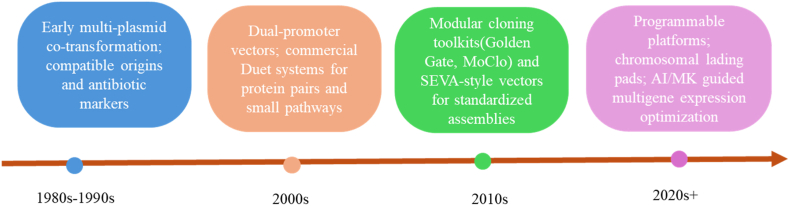
Timeline of key milestones in the development of multigene co-expression strategies in *Escherichia coli*, from early multi-plasmid approaches to modular cloning toolkits and programmable, AI-assisted expression platforms.

Over the past four decades, multigene expression strategies in *E. coli* have evolved through several distinct phases. Early work in the 1980s and 1990s primarily relied on simple multi-plasmid co-transformation, using compatible replicons and antibiotic markers to co-express a few individual genes. In the 2000s, dual-promoter vectors and commercial co-expression systems such as the pETDuet series became widely adopted, enabling more controlled co-expression of protein pairs and small pathways on a single plasmid. The subsequent decade saw the introduction of modular cloning toolkits and standardized vector architectures (e.g., SEVA-like systems), which greatly facilitated the assembly of large multigene constructs and combinatorial libraries. Most recently, the field has shifted toward programmable expression platforms that integrate modular DNA assembly, genome integration, and computational or AI-guided optimization of gene dosage. This historical trajectory is summarized in Fig. 1, highlighting the progressive move from ad hoc construct design toward systematic, platform-based co-expression engineering.

This review provides a strategic overview of multi-gene co-expression systems in *E. coli*. We compare the principal engineering architectures—including polycistronic designs (e.g. using IRES elements or 2A peptides), multi-promoter cassettes, and modular multi-vector systems—highlighting their relative advantages and limitations. We then examine key bottlenecks and design trade-offs (such as gene dosage imbalances, plasmid incompatibility, and protein folding challenges) that influence co-expression success. Next, we explore application horizons ranging from synthetic metabolic pathways and biomanufacturing of high-value compounds to the assembly of multi-protein complexes. We further discuss emerging opportunities toward programmable expression platforms, encompassing modular toolkits, automation, AI-guided design, and whole-cell modeling. Finally, we conclude with perspectives on future developments, outlining how next-generation co-expression strategies can transform *E. coli* into an even more powerful workhorse for multi-gene genetic engineering.

## 2 Engineering architectures for co-expression in *E. coli*

Co-expression strategies in *E. coli* can be categorized by how multiple genes are arranged and regulated. Broadly, one can employ multiple plasmids (vectors) versus a single plasmid to carry all genes. Within a single plasmid, genes may be organized under one promoter (polycistronic) or multiple promoters (multicistronic). Each approach comes with distinct design considerations.

### 2.1 Multi-plasmid co-expression (multiple single-gene vectors)

The most straightforward approach to co-express several proteins in *E. coli* is to transform the cell with separate plasmids, each harboring a single gene and its own expression elements [6]. This modular strategy allows each gene to be regulated independently (with its own promoter, terminator, ribosome-binding site, etc.) and often uses well-established single-gene expression vectors. It also keeps individual plasmid sizes smaller, which can improve replication and stability in the host cell [7]. In practice, multi-plasmid co-expression requires careful planning to ensure compatibility. Each plasmid must typically have a distinct origin of replication and antibiotic selection marker to be stably maintained together [8]. For example, an *E. coli* cell might carry a high-copy plasmid (ColE1 origin, ampicillin resistance) coexisting with a medium-copy p15A-origin plasmid (kanamycin resistance) and a low-copy origin plasmid (chloramphenicol resistance), each encoding a different gene. Replicon identity and plasmid copy number determine gene dosage. Typical examples include pSC101 (low copy, ∼5), p15A (medium copy, ∼10–20), and ColE1 derivatives (medium to high copy, depending on the variant). Antibiotic resistance markers (Amp/Kan/Chl) do not dictate copy number; they function solely to maintain selection.Non-antibiotic selection schemes can substitute for or complement antibiotic selection, including auxotrophic complementation (thyA, alr), toxin–antitoxin–based maintenance (ccdB/ccdA), operator–repressor systems, and metabolic antagonism, among others.Using different replicon types avoids plasmid incompatibility, where plasmids with the same origin cannot be stably inherited together [9]. Empirically, co-transformation efficiencies drop as the number of plasmids increases, and the cell's growth rate may be adversely affected by the burden of maintaining multiple replicons and multiple antibiotics in the medium [10]. Early studies recommended using two “compatible” plasmids at most, unless additional selection pressure or chromosomal integration is applied [11]. Herein, additional selection pressure refers to supplementary maintenance mechanisms beyond the primary selection marker that stabilize the co-maintenance of multiple plasmids—for example, the concurrent use of orthogonal antibiotic markers, auxotrophic complementation (e.g., thyA, alr), toxin–antitoxin (“addiction”) systems (e.g., ccdAB), or active partitioning modules (e.g., parABS). These measures reduce plasmid loss and help preserve defined vector combinations during cultivation. Chromosomal integration is not a means to enhance plasmid maintenance. Rather, it is an alternative to plasmid carriage that removes plasmid-related instability and burden when long-term genetic stability is required. In addition, carrying several plasmids in parallel can exacerbate unwanted homologous recombination events. Shared sequence features across plasmids, such as identical promoters, terminators, selection markers, or fusion tags, may recombine and give rise to plasmid rearrangements, partial or complete loss of expression cassettes, or the collapse of multiple plasmids into a single hybrid replicon. Such recombination-driven instability often results in heterogeneous cell populations and progressive loss of co-expression during prolonged cultivation. Nevertheless, multi-plasmid systems remain useful for their flexibility—one can mix and match plasmids to assemble different gene combinations—and for enabling differential control (e.g. inducible expression of one gene and constitutive expression of another).

### 2.2 Polycistronic vectors (single promoter, multiple ORFs)

An alternative to juggling multiple plasmids is to engineer a single plasmid that carries all genes as a tandem array under one promoter, producing a single multi-gene transcript (messenger RNA). Many native *E. coli* operons exhibit translational coupling, whereby a ribosome terminating on an upstream cistron reinitiates at an adjacent downstream start codon without fully dissociating from the mRNA. In engineered polycistronic vectors, this phenomenon can be intentionally leveraged to tune stoichiometry across open reading frames; however, it is not mandatory and designers typically provide an explicit RBS for each ORF to ensure predictable initiation. In practice, coupling strength can be modulated by (i) intercistronic spacing and stop–start overlaps (short or overlapping junctions, for example, TAATG or TGATG tend to favor reinitiation, whereas longer spacers dampen coupling), (ii) RBS strength and SD–start distance (commonly ∼5–9 nt), (iii) gene order (placing rate-limiting steps upstream or downstream to achieve desired ratios), and (iv) local mRNA secondary structure and stability. When decoupling is desired, insulated ribosome binding sites (RBSs) with adequate spacers and structure-minimizing junctions are used to reduce unintended carry-over of ribosomes. Collectively, translational coupling is an established, context-dependent design feature that can be exploited to balance multi-enzyme pathways in *E. coli* polycistronic constructs.This single-transcription-unit design tightly links the expression of all genes to one regulatory control, simplifying induction or repression to a single switch [12]. Polycistronic vectors tend to be smaller in total DNA content than multi-promoter constructs (since only one promoter and one terminator are needed for all genes), which can facilitate cloning and transformation [12]. Indeed, polycistronic co-expression often streamlines experimental workflows by allowing one-step transfer of multiple genes into a host via a single plasmid [13]. This approach has been effectively used to express multi-enzyme pathways and multi-subunit complexes in *E. coli*, sometimes yielding improved efficiency compared to using multiple separate expression plasmids [14]. For instance, Shenoy et al. constructed a modular polycistronic vector with tandem 2A peptide sequences to co-express several enzymes, achieving high titers of a terpene product in yeast [15]—a concept that is readily transferable to *E. coli* metabolic engineering.

In *E. coli*, canonical pathway modules are frequently expressed from a single polycistronic transcription unit with individual RBSs per ORF. A widely used example is carotenoid biosynthesis: the crtE–crtB–crtI operon (often from Pantoea/Erwinia) is assembled under a single inducible promoter to produce lycopene, with RBS strength and gene order tuned to balance flux across the three enzymes *refs*. Another well-established case is violacein biosynthesis, where vioA–vioB–vioE–vioD–vioC are encoded on one polycistronic construct, and stoichiometry is adjusted via modular RBSs and intercistronic spacing to optimize pigment titers *refs*. Additional phenylpropanoid examples include resveratrol production from p-coumarate, in which tal–4cl–sts are arranged as a single TU and tuned by RBS libraries to improve precursor channeling and product formation *refs*. Collectively, these cases illustrate that polycistronic vectors are a practical and predictable strategy for pathway incorporation in *E. coli*, provided that RBS strengths, gene order, and spacer/stop–start architecture are systematically optimized *refs*.

Despite their appeal, polycistronic systems present some design challenges. The arrangement of genes in the operon can influence their expression levels; upstream cistrons in the mRNA are often expressed more strongly than downstream ones, a phenomenon observed in both prokaryotic and eukaryotic contexts [16]. Renaud-Gabardos et al. noted that the positional effect within a polycistronic cassette can skew protein yields, necessitating gene order optimization for balanced output [17,18]. Additionally, while *E. coli* can handle long operonic mRNAs, very large inserts (many kilobases encoding multiple proteins) may destabilize the plasmid or be prone to recombination and partial loss during propagation [19]. In conventional T7-based expression systems and commonly used *E. coli* hosts, a single transcription unit typically has a practical upper limit of 4–6 ORFs or ∼8–10 kb of insert. Beyond ∼15–20 kb, the risk of recombination/deletion rises markedly, necessitating modularization across multi-promoter architectures, multiple compatible plasmids, or chromosomal integration. Thus, there is an upper practical limit to the number of genes (or total size of DNA) one can reliably include in a single transcriptional unit.

Several molecular strategies exist to implement polycistronic expression. The simplest in *E. coli* is to include a strong RBS before each open reading frame on the polycistronic mRNA, ensuring that ribosomes can initiate translation for each gene. This approach draws on *E. coli*'s natural ability to translate polycistronic operons and has been widely used epresentative examples include the dual-promoter/dual-T7 “Duet” series and related two-promoter plasmids, as well as constructs that place T7-lac and PBAD on the same backbone for coordinated control of the GABA pathway [20,21]. In more complex scenarios or in eukaryotic systems where true polycistronic translation is not native, synthetic elements like internal ribosome entry sites (IRES) and self-cleaving 2A peptides have been employed to link multiple ORFs in one transcript [22]. While highly effective in eukaryotes for cap-independent translation, IRES designs are less common in bacteria, as the native mechanism of the bacterial ribosome binding site (RBS) serves the functional role of ribosome recruitment. The core principle of IRES elements (cap-independence) is not relevant to bacteria, which lack a 5′ cap structure. Consequently, their application in *E. coli* metabolic engineering is extremely rare. The few examples of IRES-like elements studied in bacteria are often derived from viral or phage sequences [23]. Studies demonstrate that structured viral intergenic region IRES RNAs can drive translation initiation in *Escherichia coli* via ribosomal protein S1–dependent mechanisms, establishing IRES-mediated internal initiation as a bona fide, though noncanonical, strategy for gene expression control in bacteria [[23], [24], [25]]. However, IRES is generally considered less reliable than RBS tuning or 2A peptides for achieving precise stoichiometry in metabolic pathways within *E. coli*.

IRES Elements: IRES sequences are RNA motifs (derived from viruses and some cellular mRNAs) that recruit ribosomes to an internal start site, allowing translation of a downstream cistron independently of the 5′ end cap structure [26]. In a bicistronic construct, an IRES placed between Gene A and Gene B permits Gene B to be translated from the same mRNA as Gene A in eukaryotic cells [27]. However, in *E. coli*, IRES elements are generally not needed because ribosome binding can be engineered via RBS sequences. Moreover, IRES-mediated translation is a eukaryotic mechanism and tends to be ineffective in prokaryotes. Even in their native context (mammalian cells), IRES elements often result in much lower expression of the downstream gene relative to the upstream gene [28]. Due to such attenuation and potential sequence burden, IRES-based designs are less common for *E. coli* co-expression, though they are conceptually important in illustrating how multiple genes can be linked on one transcript.

2A Peptides: The 2A peptide strategy has become a popular means to achieve polycistronic expression of multiple proteins. Originally discovered in foot-and-mouth disease virus [29], 2A peptides are short (∼18–20 amino acid) sequences that cause a “ribosomal skip” during translation, resulting in the production of two separate polypeptides from one mRNA [30]. When Gene A and Gene B are connected by a 2A sequence, the ribosome translates Gene A and the 2A peptide, then fails to form a normal peptide bond at the 2A–2B junction, releasing Gene A protein with a few extra residues and continuing to translate Gene B as a new protein [31]. This yields near-equimolar expression of multiple proteins from a single open reading frame transcript [32]. In *E. coli*, 2A peptides have been successfully used to co-express enzymes in metabolic pathways, offering advantages over IRES due to their small size and more reliable cleavage [33]. For instance, Mejía-Manzano et al. utilized a 2A-linked construct to produce a plant flavonoid in yeast [34], and similar designs have been applied in bacteria to avoid the drastic drop-off in downstream gene expression associated with IRES elements [25]. The 2A strategy does leave a scar: one protein carries a few extra C-terminal residues (the “landing” from the 2A) and the downstream protein has an N-terminal proline from the 2A motif [35]. These short tags are often benign, but their effect on protein function should be considered in critical applications. Multiple distinct 2A peptides (from different viruses) can be used in tandem to link three or more genes, as shown by Szymczak-Workman et al. who constructed triple and quadruple-cistronic vectors using repeated 2A sequences [36]. Overall, 2A peptides have proven to be a powerful tool for multicistronic gene assembly in *E. coli* and other systems, due to their reliability and minimal genetic footprint.

### 2.3 Multi-promoter (multi-cistronic) vectors

Instead of a single transcript, co-expression can be achieved by constructing a plasmid with multiple independent transcription units, each with its own promoter, RBS, and terminator driving expression of one gene. In this multiple-promoter strategy, a single plasmid effectively behaves like several separate expression vectors fused together. For example, a tricistronic plasmid might have promoter1–GeneA–terminator, followed by promoter2–GeneB–terminator, and promoter3–GeneC–terminator, all in one circular DNA. Each gene is transcribed to its own monocistronic mRNA, allowing more direct control over individual gene expression levels compared to a polycistronic operon [37]. This design circumvents issues of translational coupling or gene order effects since each mRNA is separate. Multi-promoter vectors have been widely used in *E. coli* for co-expression of pathways and complexes, particularly when the expression of one component needs to be stronger or weaker relative to others. For example, Dual-T7 systems such as pETDuet, pACYCDuet, and pCOLADuet are widely used for co-expression of protein dimers and chaperone–substrate pairs [38]. Housing T7-lac and PBAD on the same plasmid enables differential induction of two pathway branches (e.g., with IPTG and l-arabinose), thereby optimizing GABA production/titers. By choosing promoters of different strengths or inducible properties, researchers can fine-tune gene dosage within the cell [21]. Zhang et al. demonstrated this approach (in *Bacillus subtilis*) by employing promoters of varying strengths for two enzymes in a redox pathway, achieving a 6.75-fold increase in product yield through balancing of expression [39]. In *E. coli*, Yao et al. similarly used a dual-promoter plasmid (T7-lac and arabinose-inducible *PBAD* on the same vector) to co-express two enzymes for GABA biosynthesis, enabling conditional control of each gene and resulting in high titers of product [40]. These examples underscore the versatility of multi-promoter systems in optimizing multi-gene workflows.

Multi-promoter vectors can be seen in two subclasses: dual-promoter systems (typically two promoters on one plasmid, often used for co-expressing two subunits or enzyme and chaperone pairs) and multi-promoter (three or more) systems for entire pathways. Dual-promoter constructs have been around since the 1980s; for instance, Müller et al. found that a plasmid with two opposing promoters expressing the HIV-1 RT heterodimer subunits yielded better assembly of the enzyme than a single orientation [41]. In industry, dual T7 promoter vectors (commercialized as the pET-Duet series) are commonly used to co-express protein pairs in *E. coli* for complex formation or multi-step biocatalysis. Moving beyond two, multi-promoter plasmids with three or more expression cassettes face increasing challenges: they become large and genetically unstable if too many inserts are included [42]. Each additional promoter and terminator consumes cloning space and can potentially interfere with neighboring promoters (promoter crosstalk or transcriptional read-through) [43]. Practically, plasmids with up to 3–4 separate expression units have been constructed and used in *E. coli*, but with diminishing returns in ease of use. He et al. reported a triple-promoter vector design, noting that careful arrangement and insulating sequences were needed to maintain stable expression of all three genes [44]. Notably, multi-promoter plasmids still rely on a single replicon—so if extremely high total gene dosage is required, engineers sometimes revert to multi-plasmid approaches or integrate some genes into the chromosome to distribute the load.

### 2.4 Modular and vector toolkit approaches

Recent trends emphasize modularity in co-expression system design. Rather than crafting each multi-gene plasmid from scratch, researchers now benefit from standardized cloning frameworks that enable rapid assembly of multiple genes. Techniques like Golden Gate assembly and modular cloning (MoClo) use sets of compatible plasmid backbones and standardized linkers to iteratively build multi-gene constructs. For example, a scientist can clone each gene into an entry vector with a chosen promoter and RBS, then combine several modules in one digestion-ligation (or Gibson assembly) step to create a multi-promoter co-expression plasmid. Such plug-and-play modular toolkits dramatically accelerate the design-build cycle for pathway construction. Additionally, dedicated co-expression vector sets have been developed: the Duet vector system (Novagen) provides a suite of *E. coli* plasmids each carrying two multiple cloning sites with different antibiotic markers and origins (e.g., pETDuet, pACYCDuet, pCOLADuet), allowing assembly of up to eight genes across four compatible plasmids in one cell. The Standard European Vector Architecture (SEVA) initiative has also generated a library of vectors where origins of replication, antibiotic cassettes, and cargo regions are interchangeable modules [45], enabling researchers to rapidly customize co-expression plasmid combinations. In practice, a metabolic pathway might be split between two plasmids (e.g., upstream enzymes on a medium-copy plasmid and downstream enzymes on a low-copy plasmid) to balance pathway flux—an approach that is inherently modular, as each “module” can be individually optimized and then combined in the final strain.

Table 1 summarizes that no single approach is universally superior—the choice depends on the specific context (number of genes, desired expression levels, and downstream application). In practice, hybrid strategies are common: for instance, two plasmids might be used, each carrying a polycistronic operon encoding part of a pathway, thereby achieving both intra-operon coupling and inter-plasmid modularity. The subsequent sections will delve into the practical challenges that arise when implementing these architectures and how researchers navigate the associated trade-offs.

**Table 1:** Conceptual comparison of co-expression strategies in ***E. coli***.

Strategy Type	Mechanism/Feature	Advantages	Limitations
**Dual Promoter**	Two independent promoters drive separate genes [4,20]	Independent expression tuning	Vector size increases; promoter interference possible
**Multicistronic Operon**	Multiple genes on one mRNA with separate RBS sites [46,47]	Simple plasmid structure; mimics prokaryotic operons	Downstream gene expression weaker; balancing needed
**Multi-vector Systems**	Genes distributed on multiple compatible plasmids [48]	Plasmid modularity; adjustable expression levels	Burden of multiple plasmids; transformation complexity
**Rare tRNA Co-expression**	Co-express rare tRNAs to improve translation [49,50]	Improved expression of rare codon-rich genes	Limited host range; tRNA imbalance may occur
**Chaperone Co-expression**	Co-express folding assistants like GroEL/GroES [51,52]	Enhanced solubility and proper folding	May not work for all targets; additional burden on cell

To complement the qualitative comparison in Table 1, we summarized several representative multigene co-expression systems in *E. coli* with basic quantitative metrics (Table 2). Across these case studies, engineered strains typically co-express 3–10 heterologous genes and reach product titers from the hundreds of mg·L^−1^ to multi-g·L^−1^ range, depending on pathway complexity and host optimization. In many examples, switching from simple single-gene or dual-plasmid setups to carefully tuned multigene architectures resulted in more than one order of magnitude improvement in titer or space–time yield. These data highlight that, when gene dosage and stoichiometry are systematically balanced, the conceptual advantages of co-expression architectures translate into substantial performance gains at the strain level.

**Table 2:** Representative multigene co-expression systems in *E. coli* and their quantitative performance.

Product (application)	Expression architecture in *E. coli*	No. of heterologous genes co-expressed	Host strain/cultivation mode	Reported titer and/or productivity∗	Design highlight (relative to baseline)	Reference
Cinnamaldehyde (nematicide, flavor compound)	Two-plasmid system: a three-gene synthetic operon (SmPAL–ScCCL–AtCCR) under the IPTG-inducible P_t_ᵣc promoter on pHB-CAD, co-expressed with a multi-gene module (aroG8/15, ydiB, aroK, pheA mutant, galP, glk) on pYHP to boost the L-Phe precursor pool.	3 pathway genes on pHB-CAD (plus 6 precursor-supply genes on pYHP)	*E. coli* YHP05 (derivative of W3110) in shake-flask cultivation	Cinnamaldehyde: 75 mg L^−1^ after 48h; 35-fold higher than W3110 (pHB-CAD) without pYHP.	Modular co-expression of a short downstream pathway with an upstream precursor-enrichment module; demonstrates that combining pathway and precursor engineering in separate plasmids can strongly enhance product titer.	[53]
(R)-α-Lipoic acid (antioxidant cofactor)	Multi-plasmid co-expression of lipoate-protein ligase A (LplA) and lipoate synthase (LipA), together with an iscSUA module to regenerate the [4Fe–4S] cluster required for LipA activity.	2 core pathway enzymes (LplA, LipA) plus 3 genes in the *iscSUA* cluster	*E. coli* BL21 (DE3) in shake-flask cultivation	LA titer increased from 20.99 μg L^−1^ (two-gene system) to 589.3 μg L^−1^ after medium optimization and co-expression of iscSUA (≈28-fold overall improvement).	Demonstrates that co-expressing auxiliary maturation machinery (Fe–S cluster biogenesis) together with the core biosynthetic enzymes can be critical for achieving high titers of cofactor-dependent products.	[54]
3-Hydroxypropionic acid (3-HP) + 1,3-propanediol (1,3-PDO) (bulk chemicals)	Multi-module co-expression of 3-HP and 1,3-PDO pathways from glycerol, combined with overexpression of transhydrogenase-based cofactor regeneration systems and deletion of competing by-product pathways.	Two full pathways (3-HP and 1,3-PDO; total >6 heterologous enzymes) plus transhydrogenase module	Engineered *E. coli* (glycerol-utilizing strain) in two-stage, pH-controlled fed-batch fermentation	Co-production of 3-HP and 1,3-PDO reached 140.5 g L^−1^ total titer with 0.85 mol mol^−1^ net yield from glycerol.	Example of high-level pathway co-expression for co-products; illustrates that balancing redox through cofactor-regeneration modules and optimizing fed-batch conditions is essential for industrial-level titers.	[55]

## 3 Bottlenecks and design trade-offs

Expressing multiple recombinant proteins in *E. coli* simultaneously poses several bottlenecks that must be addressed through thoughtful design. Key issues include achieving balanced expression of all genes, maintaining plasmid stability and compatibility, ensuring proper protein folding (and avoiding aggregation), and minimizing the metabolic burden on the host. Below we discuss these challenges and the trade-offs inherent in co-expression system design.

### 3.1 Gene dosage balancing and expression stoichiometry

One critical consideration is how to attain the right ratios of each protein. Many multi-subunit complexes or metabolic pathways require a specific stoichiometry of components for optimal function; an imbalance can lead to unused subunits, formation of misassembled complexes, or accumulation of metabolic bottlenecks. In co-expression, gene dosage is influenced by promoter strength, ribosome binding site efficiency, mRNA stability, and gene copy number (plasmid copy or genome integration). In polycistronic operons, the upstream genes often dominate expression, whereas downstream genes may be produced in lower amounts [56]. This can be.

Partially counteracted by reordering genes or using strong RBS sequences for downstream ORFs, but finding the ideal configuration may require iteration. Multi-promoter systems provide more direct control: each gene's promoter and RBS can be individually chosen or mutated to modulate its expression level [57]. For example, in a three-enzyme pathway, a strong promoter might drive the rate-limiting first step, while weaker promoters drive later steps to prevent intermediate buildup. Likewise, using plasmids of different copy numbers is a strategy in multi-plasmid co-expression—a high-copy plasmid for a protein needed in large quantity and a low-copy plasmid for a protein that is toxic or needed in smaller amounts. Achieving the correct stoichiometry often requires empirical optimization. There is a trade-off between precision and effort: more tunable systems (multi-promoter, multi-plasmid) give fine control but are more complex to construct and maintain, whereas a single operon is simpler but may need extensive trial-and-error to balance.

### 3.2 Plasmid compatibility and stability

When multiple plasmids are used, their stable maintenance in one cell is a major concern. Classical plasmid incompatibility theory holds that two plasmids sharing the same replication origin cannot coexist indefinitely in a non-selective environment because they compete for the same replication machinery and are randomly partitioned to daughter cells. Traditionally, co-expression experiments have employed plasmids with different replicon families (e.g., ColE1, p15A, RSF1030, pSC101) so that each is independently maintained [58]. Additionally, distinct antibiotic selections are applied to ensure all plasmids are retained—although this imposes a burden on the cells and can slow growth significantly with three or more antibiotics present [59]. Interestingly, recent studies suggest plasmid “incompatibility” is less black-and-white than once assumed. Velappan et al. reported that *E. coli* can stably propagate two plasmids with the same origin if strong selection is applied, at least for the timescale of typical expression experiments [60]. More recently, Mamaeva et al. systematically co-transformed *E. coli* with two ostensibly incompatible ColE1-based plasmids and found that both could be maintained together in over 75 % of cells after several days, provided antibiotic pressure was present [61]. The expression levels of each protein were determined primarily by the gene and vector sequence rather than the presence of a second plasmid [62]. Nonetheless, relying on continuous selection is necessary in those cases; if antibiotics are removed, cells rapidly lose extra plasmids to reduce burden. For multi-promoter single-plasmid designs, the stability considerations shift to plasmid size and integrity. Very large vectors (e.g., >15–20 kb inserts) can experience elevated rates of recombination or deletion, especially in rec^A^^+^ hosts. Using rec^A-^ deficient strains and optimizing insert lengths can mitigate this.

In parallel with choosing compatible replicons and antibiotic markers, several intrinsic stabilization strategies have been developed to address plasmid instability. Multimer resolution systems introduce specific recombination sites (e.g., cer or dif sites on ColE1-type plasmids) that are recognized by host-encoded recombinases and resolve plasmid multimers back into monomeric forms. This prevents accumulation of plasmid dimers or higher-order multimers, which are partitioned inefficiently and can lead to rapid plasmid loss in growing cultures. In addition, active partitioning modules (such as par or sop systems) and toxin–antitoxin “addiction” cassettes can be incorporated to ensure faithful segregation of plasmids at cell division and to selectively eliminate plasmid-free segregants, respectively. Balanced-lethal or auxotrophic complementation systems, where an essential host function is supplied only from the plasmid, offer another way to maintain plasmids without high antibiotic concentrations, which is attractive for large-scale or regulatory-sensitive processes.

In summary, genetic stability is a trade-off against architectural complexity. Distributing functions across multiple plasmids increases design flexibility but raises the risk of plasmid loss, whereas consolidating many promoters and genes on a single large vector can trigger recombination and structural instability. Combining rational plasmid architecture (compatible origins and selection markers) with dedicated stabilization modules (multimer resolution, partition systems, addiction or balanced-lethal strategies) is therefore essential for robust long-term co-expression.

### 3.3 Protein Folding and Co-expression of Chaperones

Overexpression of recombinant proteins in *E. coli* often leads to folding stress. This risk is amplified in co-expression scenarios, as multiple foreign proteins must fold simultaneously. One way to alleviate folding bottlenecks is to co-express folding helper proteins such as chaperones, foldases (e.g., disulfide bond isomerases), or specific binding partners that stabilize the target protein. For example, co-expressing cytosolic chaperones like GroEL/GroES or DnaK/DnaJ/GrpE has been shown to increase soluble production of many difficult eukaryotic proteins in *E. coli* [63]. In one study, overexpression of foldase oxidoreductases (DsbC and DsbG) alongside a target protein enhanced the correct formation of disulfide bonds and boosted active protein yield [64]. Chaperones such as Trigger Factor (TF) can be especially beneficial; Kudhair and Green demonstrated that co-expression of Trigger Factor significantly reduced aggregation of a recalcitrant protein in *E. coli*, although total yield did not fully reach native levels [65]. Notably, *E. coli* offers specialized strains (like BL21 ArcticExpress or Rosetta-gami) that come pre-equipped with extra chaperones or foldases, simplifying such co-expression needs. The trade-off in adding chaperones is the additional metabolic load—resources diverted to chaperone production—and the possibility that overactive chaperones might interact with nascent polypeptides in ways that slow their folding or lead to off-target effects. Nonetheless, in cases where inclusion bodies are a severe problem, coexpressing one or more folding assistants is often a worthwhile strategy to achieve a higher fraction of soluble, functional protein [66]. Co-expression systems thus often include at least one “auxiliary” gene (not part of the desired pathway or complex) dedicated to improving folding, processing, or export of the target proteins.

Inclusion body formation has traditionally been viewed as a negative outcome to be avoided, since aggregated proteins are inactive and require refolding steps to recover [67]. Common tactics to minimize inclusion bodies include lowering induction temperature, reducing inducer concentration, or as mentioned, co-expressing chaperones. However, recent research has started to *exploit* inclusion bodies in co-expression contexts. In some cases, intentionally sequestering one protein as an inclusion body can protect it from degradation and later allow its controlled refolding. Song et al. provided a striking example: they co-expressed a human α-lactalbumin (HLA) alongside a normally insoluble mouse cryptdin peptide in *E. coli*. The HLA preferentially formed inclusion bodies, co-aggregating with the cryptdin and shielding it from proteolysis [68]. This strategy yielded high amounts of cryptdin that could be solubilized and activated from the inclusion bodies. Thus, co-expression can be deliberately used to nucleate “protective” inclusion bodies for otherwise unstable proteins, turning a challenge into a production strategy. Generally though, unless one has such a specific plan, inclusion bodies remain an issue to mitigate. The formation of aggregates indicates a misfolding problem: the protein's folding rate is slower than its synthesis rate or it lacks necessary folding companions. In multi-gene systems, one misfolded protein can sometimes drag others with it (e.g., if two subunits are meant to assemble, one misfolded partner can cause the complex to misassemble). Hence, balancing expression (as discussed) and potentially lowering overall expression levels may be needed to avoid saturating the folding capacity of the cell. The bottom line is that solubility vs. yield is a frequent trade-off—pushing higher expression often increases the fraction of insoluble protein, so finding the sweet spot where each protein is expressed at a level the cell can fold is critical. Co-expression of solubility-enhancing fusion tags (like MBP or GST on one partner) could be another design decision to improve folding at the cost of additional steps to remove the tags later.

### 3.4 Metabolic burden and resource competition

Although *E. coli* is a resource-intensive and prone to stress responses microbe, loading it with the task of producing multiple recombinant proteins can impose a heavy metabolic burden. Transcription and translation are energy-intensive processes; co-expressing a multi-enzyme pathway can redirect a substantial portion of the cell's resources (ATP, amino acids, ribosomes) toward foreign protein production. This can slow the host's growth and reduce overall biomass accumulation, which in turn might limit total protein yield. Moreover, highly expressed proteins can compete for chaperones and secretion machinery, as discussed. In extreme cases, the accumulation of foreign protein (especially if misfolded) triggers stress responses that alter cellular physiology (e.g., heat shock response, oxidative stress if misfolded proteins accumulate in the periplasm). Excessive periplasmic expression imposes a heavy demand on disulfide-bond formation and isomerization and promotes aggregation of unfolded proteins, thereby activating oxidative and protein-folding stress as well as envelope stress responses. Co-expression of the periplasmic disulfide isomerases DsbC and DsbG, together with mild induction conditions, can mitigate these effects. Designers of co-expression systems must therefore consider the burden: Is it better to express all genes strongly at once, or to stagger expression? Some elegant systems have been developed where one gene is expressed first (or at higher level) and others later, to avoid peak load. For example, using promoters with different inducer responses can allow stepwise induction (first add IPTG to express chaperones, then arabinose to express the main enzyme, etc.). Another tactic is to use regulatable promoters and only express the pathway during a specific growth phase, allowing cells to reach high density first. The interplay between growth and production is a classical trade-off in bioprocess optimization, and it becomes more pronounced as the number of expression targets increases.

In summary, multi-gene co-expression in *E. coli* demands a holistic design approach. One must juggle gene arrangement (operon vs. individual promoters), vector choices (single vs. multi-plasmid), and supportive measures (chaperones, tuning expression) to address bottlenecks. There is often no perfect solution—improving one aspect may compromise another. For instance, adding an extra plasmid to carry a needed chaperone might solve a folding issue but introduce a plasmid compatibility headache. Experienced practitioners iterate on constructs, sometimes using combinatorial libraries of RBS or promoter variants, to empirically find a well-balanced co-expression system. Fortunately, as discussed next, the payoff for solving these challenges is substantial: multi-gene *E. coli* systems unlock a wide array of applications in biotechnology that would be impossible with single-gene expression alone.

## 4 Application horizons

Multi-gene co-expression has become an enabling technology for numerous applications in synthetic biology and biotechnology. By orchestrating complex genetic programs in *E. coli*, researchers can reconstitute whole metabolic pathways for production of valuable compounds, assemble multi-protein complexes and molecular machines, and create sophisticated genetic circuits. Here we highlight three major application areas—synthetic metabolic pathways, protein complex assembly, and biomanufacturing of high-value compounds—illustrating how co-expression systems are pushing the frontiers of what *E. coli* can do.

### 4.1 Synthetic metabolic pathways and high-value compound production

One of the most impactful uses of *E. coli* co-expression systems is in constructing synthetic metabolic pathways for biosynthesis of chemicals, fuels, pharmaceuticals, and other high-value products. Many biosynthetic pathways involve multiple enzymatic steps often sourced from different organisms. By co-expressing all the necessary enzymes in *E. coli*, one can create an efficient microbial “factory” that converts inexpensive substrates into a target product. For example, Bang et al. engineered *E. coli* to produce the flavor and fragrance compound cinnamaldehyde by co-expressing a suite of heterologous enzymes in the cinnamic acid pathway, achieving significantly enhanced titers [69]. In another study, Xiao et al. built a pathway for the antioxidant (R)-α-lipoic acid in *E. coli* through multi-gene co-expression, including enzymes for precursor supply and cofactor regeneration, resulting in high-yield production of this nutraceutical [54]. Co-expression has also enabled co-production of multiple products: Zhang et al. co-expressed an optimized set of enzymes to simultaneously synthesize 3-hydroxypropionic acid and 1,3-propanediol from glycerol in *E. coli*, balancing flux between the two branches for efficient dual production [70]. These cases exemplify how metabolic engineering relies on combining genes from various sources into one host. The co-expression system must not only express all enzymes but often do so in a tuned manner to prevent bottlenecks: an excess of upstream enzyme can cause toxic accumulation of intermediates, while a weak downstream enzyme can become the rate-limiting step. By adjusting plasmid copy and promoter strengths (as discussed in the previous section), engineers achieve the desired flux distribution. The reward is the microbial production of compounds that traditionally required costly extraction from nature or chemical synthesis. Beyond small molecules, *E. coli* co-expression has been used to produce complex biomolecules—for instance, co-expressing multiple enzymes for alginate polymer biosynthesis (an important biopolymer) allowed industrial production of alginate precursors in an *E. coli* system [71]. In the pharmaceutical realm, Pan et al. improved hydrocortisone (a steroid drug) yield by co-expressing a cascade of enzymes in *E. coli*, demonstrating the feasibility of producing complex natural products via bacterial fermentation [72]. As pathway engineering ambitions grow—such as constructing artificial metabolic pathways for new-to-nature compounds or potent drugs—multi-gene expression platforms in *E. coli* will continue to be a foundational tool.

### 4.2 Protein complex assembly and multi-subunit biologics

Many proteins of interest in medicine and research function as multi-subunit complexes. Examples include therapeutic antibodies (composed of heavy and light chains), virus-like particles (made of multiple coat proteins), membrane protein complexes, and cellular machineries like the replisome or proteasome. Co-expression in *E. coli* provides a means to produce these complexes by having all necessary subunits expressed together in the same cell, allowing them to fold and assemble in vivo. A classic success story is the co-expression of the two subunits of HIV-1 reverse transcriptase in *E. coli*, which produced an active heterodimeric enzyme—something that cannot be obtained by expressing either subunit alone [73]. Similarly, researchers have co-expressed the subunits of insulin-like growth factor binding proteins [74], hemoglobin analogs, and many other complexes in *E. coli*, enabling functional studies and easy purification of assembled complexes (often by tagging one subunit and pulling down the complex). Kerrigan et al. reviewed numerous cases where co-expression facilitated protein–protein interaction studies and complex recovery, noting that co-expressed proteins often correctly assemble and can be co-purified in a single step [75]. In structural biology, co-expression is routinely used to prepare samples for X-ray crystallography or cryo-EM—for instance, co-expressing a protein with its binding partner or chaperone can stabilize a conformation suitable for crystallization [76].

Co-expression systems have also advanced the development of biologics and vaccines. For example, to produce virus-like particles (VLPs) as vaccines, multiple viral capsid proteins are co-expressed in *E. coli* so that they spontaneously assemble into a particle that mimics the virus. *E. coli* can even be used to produce complex mammalian proteins if paired with the right partners: Bae et al. co-expressed the influenza virus hemagglutinin (HA) with a folding chaperone (caveolin-1 and an Oct1 fusion) in *E. coli*, which significantly improved the solubility and assembly of HA into particles, aiding the development of a soluble VLP-based flu vaccine [77]. Another frontier is engineering *E. coli* to co-express entire pathway complexes such as a polyketide synthase (PKS) or nonribosomal peptide synthetase—huge enzymatic complexes with multiple subunits or domains. Engineering *E. coli* to co-express entire pathway megacomplexes—such as PKSs or nonribosomal peptide synthetases (NRPSs), large modular enzymes composed of multiple subunits and/or domains—enables the heterologous reconstitution of complex biosynthetic routes [78]. Successful expression of a functional PKS often requires co-expressing several large subunits plus helper enzymes like phosphopantetheinyl transferases. With the right vector design, *E. coli* has produced complex natural products like antibiotics via heterologous PKS gene clusters, illustrating that multi-gene bacterial co-expression can even rival *Streptomyces* or other native producers.

In the realm of protein–protein interaction discovery, co-expression can be used as a screening tool. Murakami et al. developed a dual-expression screening method in *E. coli* to test pairwise combinations of calcium channel subunits, which led to the identification of a novel binding interface between two channel proteins [79]. Because *E. coli* is easy to manipulate, libraries of gene pairs can be co-transformed and screened for functional interactions (for example, by a downstream reporter or pull-down assay). This approach complements other interaction mapping techniques and can handle membrane proteins or toxic proteins better than yeast two-hybrid in some cases. The STRING database compiles known and predicted protein–protein associations, many of which are supported by co-expression evidence across organisms—highlighting that genes encoding interacting proteins often show correlated expression [80]. In synthetic biology, one might design *E. coli* circuits where multiple regulatory proteins are co-expressed to form complex logic gates or signaling cascades, expanding the toolkit of genetic circuit design beyond single-gene toggles.

### 4.3 Biomanufacturing and industrial biotechnology

*E. coli* co-expression systems are increasingly employed in industrial biomanufacturing processes to produce high-value compounds efficiently. Beyond the pharmaceuticals and fine chemicals noted earlier, this includes commodity and specialty chemicals, biofuels, and novel materials. For example, *E. coli* strains have been co-engineered to convert renewable feedstocks into biofuels like isobutanol by introducing multi-gene pathways that enable utilization of unconventional substrates. Gu et al. constructed an *E. coli* strain that can synthesize isobutanol from acetate as the sole carbon source, requiring the coordinated expression of a synthetic pathway to channel acetate into the isobutanol biosynthesis route [81]. Co-expression was key to coupling the utilization of a cheap carbon feed (acetate, possibly derived from biomass or waste) to the production pathway. In another case, co-expression of multiple enzymes enabled the conversion of lactic acid into value-added chemicals—demonstrating that *E. coli* can serve as a versatile chassis to upgrade low-value precursors to more valuable products via multi-step transformations [82]. In the food industry, metabolic pathway co-expression in *E. coli* has been used to make flavors, sweeteners, and nutritional additives. We already mentioned cinnamaldehyde (which has applications as a natural flavor and also as an insecticide in agriculture [83], and other examples include vanillin, nootkatone, and steviol glycosides, all produced by expressing multi-enzyme plant pathways in bacteria. The ability to co-express an entire plant metabolic route in *E. coli*—often involving cytochrome P450s, reductases, and other difficult enzymes—is a testament to improved vector systems and host engineering that provide needed cofactors and membrane environments for these enzymes. Furthermore, co-expression is used to improve cofactor recycling: to drive an equilibrium towards product, one might coexpress an additional enzyme that regenerates NADH or ATP in situ, thereby pushing the main pathway forward.

Another emerging area is synthetic biology materials: for example, multi-gene co-expression has enabled *E. coli* to produce spider silk analogs, collagen, and other biomaterials by providing not just the structural protein gene but also accessory proteins for post-translational modifications or proper assembly. As an illustration, to produce an elastin-like polypeptide that requires hydroxylation of prolines, *E. coli* can be co-engineered to express a prolyl-4-hydroxylase enzyme alongside the target polypeptide [21], something that has been demonstrated in recombinant systems.

Across these examples, the recurring theme is that co-expression multiplies *E. coli*'s capabilities. Instead of being limited to making one enzyme or product, a single strain can carry out multi-step processes or form multi-component structures. This significantly expands the horizon of biomanufacturing—complex pharmaceuticals, polymers, or even dynamic living materials (consortia of *E. coli* with synthetic pathways) become achievable. It is worth noting that with increasing scale and complexity, engineers must pay more attention to the interplay of the introduced genes and the host's physiology, as metabolic load and byproducts could impact yields. Hence, new tools for programmable and optimized expression—become crucial in guiding effective design.

### 4.4 Scale-up considerations for industrial co-expression

When multigene co-expression systems are transferred from shake flasks to large-scale fed-batch fermenters, additional process parameters become critical. Besides genetic stability and metabolic burden, engineers must control high-cell-density cultivation, oxygen transfer, and induction regimes to maintain reproducible expression of all pathway components. For instance, high specific growth rates at industrial scale can exacerbate resource competition, leading to plasmid loss or heterogeneous expression across the population; reducing the temperature or specific growth rate during the production phase often improves product quality and stability. Moreover, the choice and timing of inducers (e.g., IPTG, lactose, arabinose) significantly influence the balance between biomass accumulation and product formation, especially in multi-gene pathways where premature induction can overload the host.

From a process engineering perspective, key scale-up variables include dissolved oxygen and mixing (kLa), pH control, feed composition and rate, and the strategy for switching from growth to production mode. Co-expression systems that rely on multiple antibiotics for plasmid maintenance are difficult to translate directly to industrial processes, where antibiotic use is restricted; thus, antibiotic-free stabilization strategies such as auxotrophic complementation, toxin–antitoxin modules, or chromosomal integration of core pathway genes are increasingly employed. Finally, online and at-line analytics (e.g., off-gas analysis, soft sensors coupled with simple expression reporters) can be leveraged to monitor the physiological state of co-expression strains and to adjust feeding or induction set-points in real time. Integrating genetic design with these bioprocess parameters is essential for realizing the full industrial potential of multigene co-expression platforms.

## 5 Toward programmable expression platforms

As the demands on multi-gene expression systems grow, researchers are turning to new technologies and methodologies to make co-expression in *E. coli* more predictable, tunable, and high-throughput. The future of co-expression is trending toward *programmable expression platforms*—integrated toolkits and workflows that allow rational design of complex genetic programs with minimal trial-and-error. Several converging advances are driving this progress: modular DNA assembly standards, automated strain engineering, computational modeling, and AI-guided optimization. Here we outline how these developments promise to revolutionize the construction of co-expression systems.

### 5.1 Modular expression toolkits

The concept of modularity has already been touched upon in the context of vector design, but it is being extended further to standardized expression platform toolkits. These toolkits comprise libraries of promoters, RBSs, terminators, and vector backbones that have defined, interoperable interfaces. One example is the Modular Cloning (MoClo) system which uses Golden Gate assembly to snap together multiple transcription units in a single tube reaction, following a standardized syntax [84]. Another example is the aforementioned SEVA plasmid collection which provides a menu of origins (high, medium, low copy), antibiotic markers, and payload modules that can be mixed and matched [45]. By using such toolkits, a researcher can design a multi-gene construct in silico, selecting parts from a registry, and then physically assemble the DNA in a rapid, reliable way. The parts are often well-characterized—for instance, a toolkit might offer a set of promoters covering a 100-fold range of strengths, each measured and assigned a “ribosome binding strength” score. This enables more quantitative predictability in expression. In essence, *E. coli* co-expression is shifting from an ad hoc art to a plug-and-play engineering discipline. Programmable plasmid systems are also emerging, such as circuits that automatically adjust the expression of one gene based on the level of another via synthetic regulatory feedback loops. In the future, we can envision a *E. coli* strain with a modular genomic platform: pre-inserted landing pads in the chromosome could accept large pathway modules via site-specific recombination (bypassing plasmids altogether for stability), and those modules could be turned on/off or modulated by programmable regulators (like CRISPR-based activators/repressors that are themselves modularly assembled). All these modular strategies aim for greater control and ease of use, so that building a new co-expression strain becomes as straightforward as writing a piece of code using standard libraries.

### 5.2 Automation and high-throughput engineering

As the complexity of co-expression designs increases (with dozens of genetic parts needing optimization), manual iterative experimentation becomes a bottleneck. Enter laboratory automation and high-throughput workflows. Forward-looking laboratories and biofoundries are employing automated pipelines for Design–Build–Test–Learn (DBTL) cycles in strain engineering. In such a pipeline, once a set of gene parts and target outputs are defined, robots can assemble combinatorial libraries of co-expression plasmids (for example, varying promoter strengths or gene orders) in microplate format. Automated transformation and cultivation systems then screen these variants for desired performance metrics (e.g., product titer, enzymatic activity, growth rate). Using multi-parallel fermentations with online sensors, dozens or hundreds of co-expression strains can be characterized in the time it once took to examine a handful. For instance, to optimize a five-enzyme pathway, an automated approach might construct a library of plasmids exploring various expression level combinations (using a factorial design of different RBSs for each enzyme), then assay each variant's output. The data can be fed into machine learning models to identify the optimal combination or to train an algorithm on how each gene's expression correlates with performance. Automation thus greatly accelerates the identification of balanced co-expression systems and can uncover non-intuitive solutions that a researcher might not test by manual intuition alone.

Furthermore, automation extends to the fermentation and scale-up stages. Co-expression strains often need careful tuning of induction timing and conditions; automated bioreactors can run different feeding or induction schedules to find the best regimen (for example, induce gene A first, then gene B an hour later, etc.). Microfluidic devices are even being explored to cultivate single *E. coli* cells or droplets with different co-expression constructs to rapidly assess phenotypes. Overall, automation reduces the human labor and variability in constructing complex expression systems, making the process more reproducible and scalable. As co-expression moves from the lab to industry, such consistency is key.

### 5.3 AI-guided design and optimization

Hand-in-hand with automation is the rise of artificial intelligence (AI) and machine learning (ML) for guiding genetic design. AI can assist co-expression system development at several levels.

Sequence design: Machine learning models can predict the strength of promoters or RBSs or the impact of codon usage on translation efficiency, allowing for in silico tuning of expression elements before any wet lab work. For example, a researcher can use an RBS calculator [85,86] or deep-learning model to design a set of RBS sequences that produce a gradient of translation rates for each gene, rather than relying purely on trial-and-error or standard parts [87,88].

Predictive modeling: Given a certain multi-gene network, AI algorithms can help predict metabolic fluxes or identify which enzyme is likely to bottleneck the pathway, thus suggesting where to allocate higher expression. Techniques like Bayesian optimization have been applied to metabolic engineering, where an algorithm proposes new genetic variants to test (combining different expression levels) based on past results, efficiently searching the design space for optimal solutions [89,90].

In practice, several concrete AI-based tools have already been applied to *E. coli* multigene systems. The Ribosome Binding Site (RBS) Calculator and related Genetic Systems Calculator provide thermodynamics-based and machine learning–augmented predictions of translation initiation rates, enabling rational tuning of RBS strengths across entire operons or multi-promoter constructs before any wet-lab work [91]. Building on such design tools, Jervis et al. implemented a machine-learning-guided design–build–test–learn cycle to optimize a nine-step mevalonate pathway for (S)-limonene production in *E. coli*, using combinatorial RBS libraries and predictive models to identify high-performing RBS combinations and achieving a substantial increase in product titer compared with heuristic designs [92]. More generally, active-learning workflows such as METIS allow users to explore high-dimensional design spaces of promoter/RBS/operon configurations with relatively few experiments by iteratively proposing the most informative genotypes to test [93]. As these AI platforms become more tightly integrated with automated DNA assembly and high-throughput screening, multigene co-expression in *E. coli* is likely to transition from a largely empirical exercise to a data-driven, model-informed engineering practice.

AI can analyze large datasets from co-expression experiments (e.g., transcriptomics, proteomics of engineered strains) to pinpoint issues such as unexpected gene silencing, metabolic imbalances, or toxic intermediates. For instance, if a co-expression strain isn't performing, transcriptome data might reveal that one gene isn't transcribed well; AI models could correlate such issues with sequence features or suggest alternative regulatory parts. Already, there are examples of using neural networks to design synthetic promoters that minimize crosstalk in multi-gene circuits, and to predict protein solubility improvements via sequence modifications—tools highly relevant to co-expression success [[93], [94], [95]].

Another exciting area is AI-driven protein engineering within co-expression. imagine using AI to design a chaperone specifically optimized to assist a particular target protein, and then co-expressing that pair in *E. coli* [96,97]. Generative AI models could propose novel chaperone sequences or synthetic protein partners to enhance yield, which could then be tested through co-expression [98]. In short, AI is becoming a powerful ally, turning the traditionally heuristic process of strain engineering into a more computationally guided exercise.

### 5.4 Whole-cell modeling and systems integration

Comprehensive computational models of *E. coli* are being developed to simulate how multi-gene interventions affect the cell. By plugging a prospective co-expression pathway into a whole-cell model, one could predict, for example, that co-expressing enzyme at high level will severely drain cellular ATP or an essential precursor, suggesting the need to also upregulate a parallel pathway or to supplement the medium [99]. Whole-cell modeling also aids in tuning expression: it can reveal, for instance, that making 10 % of total protein as enzyme A and 5 % as enzyme B yields the highest flux to the product, which then guides the selection of promoters that achieve those protein fractions [99]. Although these models are complex and not yet perfectly accurate, they are continually improving with more experimental data. They represent a move towards a *rational design* paradigm, where one can virtually test a co-expression strategy on a “digital twin” of *E. coli* before constructing it in the lab.

Beyond metabolism, systems biology models are used to predict the outcomes of regulatory circuits involving co-expressed transcription factors or signaling proteins. Synthetic biologists are designing *E. coli* strains where multi-gene networks behave like logic gates or dynamic oscillators; modeling is essential to ensure these networks function as intended once physically implemented [[100], [101], [102]].

Looking further ahead, the integration of these approaches—modular toolkits, automation, AI, and modeling—points to an era of fully programmable biological factories. We might soon see cloud-based platforms where a user specifies a metabolic product or a protein complex, and the platform's algorithms automatically design an *E. coli* strain with the required co-expression system, which is then built by robotics. The strain would be pre-optimized to minimize burden and maximize yield, using modeling to avoid pitfalls and AI to refine the design. In effect, co-expression in *E. coli* is on the path from an experimental science to an engineering discipline supported by computational power and high-throughput capabilities.

## 6 Concluding perspectives

The evolution from single-gene expression to sophisticated multi-gene co-expression systems marks a significant leap in our ability to program biology. *E. coli*, as a long-standing workhorse, has proven remarkably adaptable to this evolution—from carrying one recombinant gene to serving as a host for entire synthetic operons, pathways, and multi-protein assemblies. This transition has enabled breakthroughs in producing complex molecules and unraveling biological mechanisms that would be unattainable with one gene at a time.

There is a diverse toolkit of co-expression architectures available (IRES-linked cistrons, 2A polycistronic chains, multi-promoter plasmids, multi-plasmid sets), each with its own strengths. Successful implementation often requires a tailored approach, possibly combining strategies to suit the specific multi-gene task. Balancing expression is both critical and challenging—too often, initial co-expression attempts falter due to one component being over- or under-produced. Advances in vector design and part libraries are making it easier to tune expression levels, but an element of empirical optimization remains. Co-expression invariably introduces trade-offs: what simplifies experimental steps might complicate cellular burden, and maximizing one protein's yield might compromise another's folding. Effective design means anticipating these trade-offs and finding acceptable compromises (for instance, a slightly lower expression level that prevents inclusion bodies may give higher functional yield overall). The payoff for mastering co-expression design is immense—from green manufacturing of chemicals to novel therapeutics and vaccines, multi-gene engineered *E. coli* are at the forefront of biotechnological innovation. We are seeing *E. coli* not just as a producer of single proteins, but as a flexible chassis that can be loaded with whole metabolic circuits or machine-like protein assemblies to carry out complex tasks.

The field is poised for further transformation by embracing computational and systems-driven methodologies. Future developments likely will focus on making co-expression systems more *predictable, robust*, *and user-friendly*. This entails developing even more comprehensive libraries of modular parts, creating standardized chassis strains optimized for multi-gene stability, and implementing global transcriptional control systems that can toggle entire pathways on/off as needed. The convergence with machine learning and automation means that we will accumulate large datasets on what works and what doesn't in co-expression, feeding back into better design rules. In time, the process of strain engineering that once took months for a team of scientists could be achieved in days by an integrated platform.

Another exciting prospect is cell-free co-expression systems (e.g., cell-free protein synthesis using *E. coli* extracts) where multiple genes can be expressed simultaneously in vitro. These could serve as a rapid prototyping tool for testing multi-enzyme reactions or multi-subunit complexes before committing to creating a stable co-expression strain. Innovations in encapsulating cell-free systems or co-culturing different engineered *E. coli* strains (each expressing a subset of genes) could circumvent some challenges of putting all genes in one cell.

In conclusion, *E. coli* multi-gene co-expression technologies have matured from a niche capability to a central role in biotechnology. The journey from monogenic to polygenic systems has vastly expanded the scope of achievable biological engineering projects. By continuing to refine our strategies and embrace new technologies, we edge closer to truly programmable microbial factories—where *E. coli* can be given a genetic “program” of arbitrary complexity and execute it with reliability. The coming years will undoubtedly bring co-expression systems that are more powerful, smarter, and easier to deploy, opening doors to innovations we can only begin to imagine today. The strategic integration of engineering principles with biological insight will guide this progression, ensuring that the next generation of co-expression platforms will turn bold scientific visions into tangible realities.

## CRediT authorship contribution statement

**Rui Liu:** Writing – review & editing, Writing – original draft. **Lu-Wei Wang:** Writing – review & editing, Writing – original draft, Resources. **Zi-Han Gao:** Resources. **Xiao-Tong Sun:** Resources. **Shu-Ran Lv:** Resources. **Huan Liu:** Writing – review & editing. **Sa-ouk Kang:** Conceptualization. **Bo Sun:** Writing – review & editing, Conceptualization.

## Funding

No financial support was received for this study.

## Declaration of competing intereset

The authors declare that they have no known competing financial interests or personal relationships that could have appeared to influence the work reported in this paper.
